# Bacteriology of Sputum Samples: A Descriptive Cross-sectional Study in a Tertiary Care Hospital

**DOI:** 10.31729/jnma.4807

**Published:** 2020-01-31

**Authors:** Bijendra Raj Raghubanshi, Bal Man Singh Karki

**Affiliations:** 1 Department of Microbiology, KIST Medical College and Teaching Hospital, Gwarko, Lalitpur

**Keywords:** *antimicrobial drug resistance*, *culture*, *respiratory tract infections*, *sputum culture*

## Abstract

**Introduction:**

Lower respiratory tract infection is a common infection and accounts for a greater burden of disease worldwide. It is a great challenge to the clinician and still more, with increasing antimicrobial resistance. Its empirical treatment may vary according to the type of causative organisms. The objective of this study is to identify the pathogenic microorganisms and their antimicrobial susceptibility pattern from sputum sample.

**Methods:**

This descriptive cross-sectional study was conducted in KIST Medical College and Teaching Hospital from February 2015 to January 2016. Ethical approval was taken from institutional review committee prior to the study with reference no. 0051/2014/15. Data on culture and sensitivity of isolates from sputum samples were collected from the records of the hospital. Sample collection, processing, identification of microorganisms and antimicrobial susceptibility tests were performed according to the Clinical and Laboratory Standards Institute guidelines. All the data were tabulated in an Excel sheet and analyzed using SPSS version 20.

**Results:**

Out of 2318 samples, 694 (29.93%) sputum samples at 95% confidence interval (737.21650.79) were reported as culture positive. Klebsiella was the most common isolate followed by Pseudomonas, Escherichia coli, Acinetobacter, Staphylococcus aureus, Candida albicans, Streptococcus pneumoniae, Streptococcus pyogenes, and others. Imipenem and vancomycin showed the most sensitivity towards gram-negative and gram-positive bacteria respectively.

**Conclusions:**

Proper diagnosis, identification of causative agents and their antimicrobial susceptibility pattern are important steps to limit the irrational use of antimicrobials. Prescribing antimicrobials empirically in the case of suspected lower respiratory tract infection is difficult.

## INTRODUCTION

Lower respiratory tract infections (LRTIs) are common infections in the general population, however, older individuals and patients with chronic diseases or compromised immune function are prone to these infections. LRTIs account for a greater burden of disease worldwide than ischaemic heart disease, cancer, malaria or human immunodeficiency virus infection so managing patients with LRTIs is a great challenge to a clinician.^1^

Different societies like the British Thoracic Society (BTS) and Infectious Disease Society of America (IDSA) have formulated the main guidelines on managing LRTIs.^2,3^ The identification of pathogenic organisms causing LRTIs is very important for treatment.^3^ The most common pathogens isolated from sputum samples are Klebsiella pneumoniae(39.5%) followed by Pseudomonas(25%), Escherichia coli (11.5%), Staphylococci (11.5%) and others (3.8%).^4^ In comparison to infections caused by susceptible bacteria, increased morbidity and mortality are associated with infections caused by resistant bacteria.^5,6^ Infections caused by resistant bacteria led to prolonged hospital stays, increased health care costs and in many cases untreatable infections.^7^

Thus, this study was conducted to identify the pathogenic microorganisms and their antimicrobial susceptibility pattern from sputum sample.

## METHODS

This descriptive cross-sectional study was done at the Department of Microbiology of KIST Medical College and Teaching Hospital. Ethical approval was taken from the institutional review committee of KISTMCTH prior to the study with reference no. 0051/2014/15. It was conducted from February 2015 to January 2016. Laboratory records from Clinical laboratory services of KIST Medical College and Teaching Hospital were reviewed for the period of 2011 July to 2015 June. All the sputum samples submitted for culture and sensitivity from patients presenting in outpatient departments and inpatient departments who were suspected of having LRTIs were included in the study. Contaminants growths were excluded.

Convenient sampling was done and the sample size was calculated using the formula,

$$\begin{array}{ll} \text{n} & \text{= }{\text{Z}}^{\text{2}}\times (\text{p}\times \text{q)}/{\text{e}}^{2}\\ & \text{= }{(\text{1.96)}}^{\text{2}}\times \text{0.5}\times \text{(1}-\text{0.5)}/{(\text{0.03)}}^{\text{2}}\\ & \text{= }0.9604/0.0009\\ & =1067.11\\ & =1068 \end{array}$$

where,

n = required sample size
p = prevalence of bacterial isolation (50%)
q = 1-p
e = margin of error, 3%
Z = 1.96 at 95 % CI

The calculated minimum sample size was 1068. As convenient sampling was done, the sample size was doubled to 2136, however, the total sample taken was 2318.

In routine clinical laboratory services processing, samples were cultured in five percent blood agar and MacConkey agar and chocolate agar. Inoculation was done with the help of a nichrome wire loop. Blood and MacConkey agar were incubated aerobically at 37°C for 48 hours. Chocolate agar was incubated at 37°C in a candle jar. The growth of mixed organisms without any predominant growth of a particular organism was reported as normal flora. The sputum samples yielding significant pathogenic organisms were reported as positive cultures. Bacterial identification was done by colony morphology, gram staining, and standard biochemical tests.^8^ Antimicrobial susceptibility test was performed by Kirby-Bauer disc diffusion technique. Different antimicrobial panels were used for different groups of microorganisms and second-line antimicrobials were used only when necessary following the Clinical and Laboratory Standards Institute (CLSI) guidelines.^9^ All the data were tabulated in an Excel sheet and analyzed using SPSS version 20.

## RESULTS

A total of 2318 sputum culture samples were included for analysis. Out of 2318 samples, 694 (29.93%) sputum samples were reported at 95% confidence interval (737.21-650.79%) as culture positive. Out of the 2318 sputum culture reports analyzed, significant pathogens were not isolated in 1624 samples. Multiple pathogens were obtained from 15 samples.

More than 18 different types of microorganisms were isolated. The most common organism isolated from the sputum sample in this study was Klebsiella species 176 (24.82%). Subsequently other common organisms were Pseudomonas 139 (19.6%), Escherichia coli 110 (15.5%), Acinetobacter 66 (9.3%), Staphylococcus aureus 63 (8.88%), Candida albicans 48 (6.77%), Streptococcus pneumonia 23 (3.24%), Streptococcus pyogenes 20 (2.82%)and others non-albicans (Candida, Citrobacter, Enterococcus, Enterobacter, Proteus, Haemophilus, Nocardia, etc).

Klebsiella was found to be the most common microorganism in all age groups while other microorganisms also followed a similar pattern (Table 1).

**Table 1:** Relationship between the age of the patient and the type of organism isolated.

Age	No. of sample processed	Organisms isolated									
		Klebsiella	Pseudomonas	Escherichia coli	Acinetobacter	Staphy lococcus aureus	Candida albicans	Streptococcus pneumonia	Streptococcus pyogenes	Others	Total
<15	24	2	2		1	1	-	-	2	-	8
15-40	604	46	34	27	14	17	3	5	3	8	157
41-60	667	56	48	30	16	16	18	9	9	16	218
>60	1023	72	55	53	35	29	27	9	6	40	326
Total	2318	176	139	110	66	63	48	23	20	64	709

None of the antimicrobials had 100% efficacy except vancomycin. However, it was used for gram-positive cocci only. For gram-negative organisms, imipenem had the maximum efficacy of 84.33% against all isolates and 86.9% against the most common organism (Table 2). Out of tested isolates, most of the organisms were found to be sensitive to Amikacin (79.66%), followed by Nitrofurantoin (76.66%) and Gentamicin (70.49%) whereas Ampicillin and Nalidixic acid had efficacy of 16.66% and 22.22% only.

**Table 2:** Antimicrobial susceptibility pattern of overall microorganisms.

Antimicrobials	No. of isolates tested (%)	No. of sensitive isolates (%)	No. of intermediate isolates (%)	No. of resistant isolates (%)
Amikacin	288 (100%)	207 (71.87)	-	81 (28.12)
Ampicillin	409 (100%)	61 (14.91)	4 (0.97)	344 (84.1)
AMC	224 (100%)	16 (7.15)	-	208 (92.85)
Carbenicillin	92 (100%)	64 (69.56)	2 (2.17)	26 (28.26)
Cefazolin	331 (100%)	52 (15.7)	6 (1.81)	273 (82.47)
Cefepime	173 (100%)	27 (15.6%)	-	146 (84.39)
Cephotaxime	346 (100%)	158 (45.66)	10 (2.89)	178 (51.44)
Ceftriaxone	219 (100%)	124 (56.62)	1 (0.45)	94 (42.92)
Ceftazidime	105 (100%)	68 (64.76)	1 (0.95)	36 (34.28)
Ciprofloxacin	573 (100%)	371 (64.74)	5 (0.87)	197 (34.38)
Cotrimoxazole	394 (100%)	202 (51.26)	-	192 (48.73)
Eryhromycin	100 (100%)	62 (62)	2 (2)	36 (36)
Gentamycin	596 (100%)	423 (70.97)	11 (1.84)	162 (27.18)
Imipenum	83 (100%)	70 (84.33)	2 (2.4)	11 (13.25)
Meropenum	153 (100%)	100 (65.35)	3 (1.96)	50 (32.67)
Ofloxacin	236 (100%)	93 (39.4)	1 (0.42)	142 (60.16)
Tobramycin	110 (100%)	93 (84.54)	1 (0.9)	16 (14.54)
Piparacillin	88 (100%)	49 (55.68)	1 (1.13)	38 (43.18)
Vancomycin	65 (100%)	65 (100)	-	-

## DISCUSSION

This study was conducted to find out the pattern of microorganisms isolated from sputum of suspected LRTI patients and susceptibility of the isolates toward different antibiotics. Culture positive rate was 29.93% in this study. A similar rate was shown by Oberoi A et al. (32%), whereas a slightly higher rate was shown in the studies by Asati RK et al. (40.64%), Edirisinghe LU et al. (56.56%).^4,10,11^ In 70.07% no pathogenic organisms were reported; this might be due to reasons like prior use of antimicrobials, infection by atypical microorganisms, viral or fungal infections or some other conditions. And if we see the isolation rate in different age group there is no much significant differences. It was 33.33% in <15 years age group and similarly 25.49% in 15-40 years, 31.93% in 41-60 years and 31.18% in >60 years age groups. In this study, the most common organisms are Klebsiella pneumoniae followed by Pseudomonas. Similar patterns of organisms were shown in the study done by Asati et al.^4^ Similarly study done by Edirishinghe LU et al. also showed Coliforms and Pseudomonas as the two most common organisms.^11^ But studies done by Oberoi A et al. showed Streptococcus pneumoniae, Pseudomonas aeruginosa as the two most common organisms.^10^ The reason behind this may be Oberoi et al. did a study in community-acquired pneumonia and Streptococcus pneumoniae is the most common organism of community-acquired pneumonia. But in our study, only 23 Streptococcus pneumoniae were isolated. It may be due to this study is not only restricted to community-acquired pneumonia and another reason maybe most of the patients may have had antibiotics before coming to the hospital and Streptococcus pneumoniae being sensitive to those antibiotics.

In antimicrobial susceptibility testing, Vancomycin was observed to be 100% effective but this is only used for gram-positive bacteria but the most common organisms are gram-negative in case of RTIs. So Vancomycin cannot be recommended as empiric treatment in LRTIs but highly effective in case of Gram-positive bacteria. Similarly, in a study done by Oberoi et al, Vancomycin seemed to be 92.7% effective.^10^ Other effective antimicrobials shown to be Tobramycin 84.54%, Imipenum 84.83%, Amikacin 71.87%, Gentamicin 70.97%. Similarly, Asati RK reported Amikacin to be 92.7% effective and Gentamicin 41.3%.^4^ Other antimicrobials like Ceftazidime, Ciprofloxacin, Ceftriaxone, Cotrimoxazole seemed to be moderately effective having efficacy rate of 64.76%, 64.74%, 56.62% and 51.26% respectively. Most of the organisms isolated showed highly resistant to Amoxycillin-Clavulanic acid (92.85%), Ampicillin (84.1%), Cefazolin (82.47%) and Ofloxacin (60.16%).

Klebsiella, the most common isolate also showed high sensitivity to Imipenem (86.9%), Amikacin (83.63%) and Gentamicin (71.51%) and high resistance to Ampicillin (97.36%), Amoxycillin-clavulanic acid (97.53%%). Ciprofloxacin (71.6%). Cotrimoxazole (58.14%), Ceftriaxone (58.92%), Cefotaxime (52.89%) were moderately effective. A study done by Shilpa K, et al. showed 66.66%, 62.50% and 56.66% sensitivity towards Amikacin, Gentamicin, and Imipenum respectively.^12^ The second most common organism Pseudomonas, also an important causative agent for nosocomial infection, was found to be sensitive to Imipenem, Amikacin, Ciprofloxacin, Gentamicin. Cephalosporin group like Cefepime (57.14%), Ceftazidime (53.92%), Cefotaxime (41.17%) were relatively less effective against Pseudomonas. Staphylococcus were highly resistant to Cephazoline (88.88%), Cefotaxime (61.76%), Ceftriaxone (50%), Ceftazidime (100) whereas sensitive to Vancomycin (100%), Amikacin (72.73%), Imipenem(66.67%) and Gentamicin(63.64%). Streptococcus pneumoniae was highly sensitive to most of the antimicrobials used. 100% of isolated Streptococcus pneumoniae were sensitive to Cefotaxime, Ceftazidime, Ceftriaxone, Ofloxacin, Gentamicin. Efficacy of Ciprofloxacin was 95%. In our region, most of the patients have the habit of coming to the hospital only after taking antibiotic and not being cured. This could be one of the reasons behind less number of Streptococcus pneumoniae isolation. Limitations of the study are: this is a retrospective study, only those antimicrobials which were tested were included, all the microorganisms were not tested against all the antimicrobials, only selected antimicrobials were tested during routine tests.

## CONCLUSIONS

In case of suspected LRTIs, we have to think a lot to prescribe antimicrobials empirically. Proper diagnosis, identification of causative agents and their antimicrobial susceptibility pattern are important steps towards limiting the irrational use of antimicrobials. This study along with other related studies are sufficient to say that most of the isolated organisms are highly resistant to most of the antimicrobials. The types of bacteria isolated and their antimicrobial susceptibility pattern may be different from place to place and time to time.
